# Validation of a health administrative definition of obstructive sleep apnea in children in Ontario, Canada

**DOI:** 10.1371/journal.pone.0347148

**Published:** 2026-04-27

**Authors:** Dhenuka Radhakrishnan, Tetyana Kendzerska, Sherri Katz, Henrietta Blinder, Saadoun Bin Hasan, Tanvi Naik, Eric Benchimol, Indra Narang, Suhail Al-Saleh

**Affiliations:** 1 Children’s Hospital of Eastern Ontario Research Institute, Ottawa, Ontario, Canada; 2 Department of Pediatrics, University of Ottawa, Ottawa, Ontario, Canada; 3 ICES, Ontario, Canada; 4 Department of Medicine, University of Ottawa, Ottawa, Ontario, Canada; 5 Ottawa Hospital Research Institute, Ottawa, Ontario, Canada; 6 Hospital for Sick Children, Toronto, Ontario, Canada; 7 Department of Pediatrics, University of Toronto, Toronto, Ontario, Canada; Xuzhou Central Hospital, The Xuzhou School of Clinical Medicine of Nanjing Medical University, CHINA

## Abstract

Obstructive sleep apnea (OSA) affects approximately 5% of children and requires polysomnography (PSG) for diagnosis. As such, population-based longitudinal studies of OSA in children are scarce. We aimed to validate pediatric case definitions of OSA using provincial health administrative datasets and establish a methodology for future longitudinal studies in this population. We performed a multicenter validation study, linking Ontario health administrative data with clinical data for children aged 0−18 years who underwent PSG between 2009–2016 at two tertiary care children’s hospitals. We used various administrative case definitions to identify those with the highest sensitivity, specificity, positive and negative likelihood ratios for capturing children with moderate-severe OSA (apnea-hypopnea index ≥5), stratified by age groups (<10 and age ≥ 10 years). The reference cohort included 1,254 children who underwent PSG, with 317 moderate-severe OSA. The overall mean age was 7.7 ± 4.8 years, with 64.8% < 10 years of age and 44% female. The best-performing case definitions included combinations of PSG and adenotonsillectomy or initiation of positive airway pressure therapy within 0−18 months post PSG, or PSG and a diagnostic code for OSA within 6 months pre and 0−18 months post PSG. In children <10 years, these definitions exhibited high specificity (0.80–0.88), with moderate sensitivity (0.62–0.79), positive (3.82–5.25) and negative likelihood ratios (0.27–0.43). However, the performance in children ≥10 was less robust with high specificity (0.85–0.92), moderate positive likelihood ratios (3.48–4.19) and low sensitivity (0.28–0.59), and negative likelihood ratios (0.48–0.78). We have identified case definitions within Ontario health administrative data that have good specificity, and moderate sensitivity, positive and negative likelihood ratios for identifying moderate-severe OSA in children <10 years of age. These can be used in future studies to understand the natural history, predictors and outcomes of young children with OSA, while refined case definitions may still be needed for children ≥10.

## Background and rationale

Obstructive sleep apnea (OSA) is the limitation of airflow during sleep caused by upper airway collapsibility leading to intermittent partial or complete obstruction. OSA has a prevalence of at least 5% in childhood [1,2], with higher rates among children with underlying comorbid health conditions. The prevalence of OSA is rising [1,2] and if left untreated, has the potential for causing significant morbidity, including neurocognitive, cardiovascular, and metabolic complications [1,3]. OSA has also been shown to be associated with increased health care use by children [4–6]. OSA is difficult to diagnose using history and physical examination alone, particularly in children [7–9]. As such, polysomnography (PSG) is the gold standard for diagnosis [10]. There is a scarcity of sleep laboratories across Canada that can diagnose OSA in children [11,12]. In the province of Ontario, the largest number of PSGs for children <10 years are performed at the Children’s Hospital of Eastern Ontario (CHEO) in Ottawa and the Hospital for Sick Children (SickKids) in Toronto; together these two centers perform approximately 1600 PSGs/year, accounting for 40% (in 2009) to 25% (in 2016) of all PSG’s in children up to 18 years of age.

The primary cause of OSA in children under the age of 10 years is enlargement of adenoid and tonsillar tissues which grow in size between 4–10 years of age before they start to naturally involute [13]. Adenotonsillectomy (AT), or the surgical removal of enlarged adenoids and/or tonsils is the recommended first line treatment for moderate-severe pediatric OSA with demonstrated efficacy [14–17]. OSA in older children (≥10 years of age) has a different pathophysiology that is more akin to adult OSA and is often driven by obesity [18,19]. In this age group the primary interventional treatment is the nocturnal application of continuous or bi-level positive airway pressure (CPAP/BiPAP) through the nose or nose and mouth via a tightly fitting mask [3].

Health administrative data collected by government or funding agencies for the purposes of tracking health care programs and services is often used for research in a broad range of health care issues. In Ontario, the availability of robust population-based administrative data that includes nearly all individuals in the province provides a resource for addressing some of the current gaps in our understanding of the burden of OSA in children and its long-term outcomes on both patients and the health care system, whereas such studies are already being conducted in adults with OSA [20,21]. A key challenge in using health administrative data for research is that diagnosis and billing codes alone do not guarantee accurate identification of children with OSA. The aim of the current study was to develop and validate an algorithm that would accurately identify children with OSA using combinations of various health care contact codes available in Ontario administrative data. As no such algorithm currently exists for pediatric age groups, this could set the foundation for future population-based research on childhood OSA. Developing and validating such algorithms is essential to ensure that subsequent research using health administrative data can accurately capture children with true OSA, so that research findings can be directly translated to inform clinical care.

Developing and validating such algorithms is essential to ensure that subsequent studies using administrative data can **accurately capture children with true OSA**, thereby strengthening the validity of research findings and enhancing their **translation into clinical care**.

## Methods

### Study design and setting

We performed a retrospective multi-center validation study of case ascertainment algorithms for pediatric OSA in children who underwent polysomnography at one of two tertiary care, academic pediatric centers: the Children’s Hospital of Eastern Ontario (CHEO), in Ottawa, Canada, or the Hospital for Sick Children (SickKids), in Toronto, Canada. Clinical sleep databases at each of these centers were linked to Ontario population-based health administrative data housed at ICES. This study was approved by the institutional research ethics boards at both CHEO and SickKids and through a privacy impact analysis at ICES. This study was reported per the Reporting of studies Conducted using Observational Routinely collected Data (RECORD Checklist) which is extended from the Strengthening the Reporting of Observational Studies in Epidemiology (STROBE) Statement (Table in S1 Table). This study was approved by ICES’ Privacy and Legal Office and by the Children’s Hospital of Eastern Ontario research ethics board (CHEOREB, approval# 20/07PE). Consent was not obtained as data were analyzed anonymously.

### Data sources

The CHEO sleep database is a clinical database that contains information on all children 0–18 years of age who underwent a PSG at CHEO between April 1, 2009 – March 31, 2012. The CHEO sleep database includes granular information on results of the PSG, sleep diagnosis, treatments the child has received and comorbid health conditions. All PSGs performed at CHEO were scored by pediatric-trained sleep technologists using American Academy of Sleep Medicine (AASM) criteria and interpreted by one of two CHEO pediatric sleep physicians. This data was accessed and collected between 15 Jan 2015–30 December 2015 and authors did have the ability to identify individual participants during data collection.

The SickKids sleep lab database is a clinical database that contains information on all children 0–18 years of age who underwent a PSG at SickKids in 2009 and 2016, including results of the PSG, sleep diagnosis, and treatments the child received. All PSGs performed at SickKids were scored by pediatric-trained sleep technologists using AASM criteria and interpreted by one of the SickKids sleep physicians. Of note, AASM criteria for scoring pediatric sleep studies was revised in 2012 and these new criteria were used to score the PSGs for in children tested in 2016 [22]. This data was accessed and collected between 15 Dec 2019–15 May 2020 and authors did have the ability to identify individual participants during data collection.

Health administrative data was securely and confidentially accessed through ICES, a non-profit organization that provides access to most of the administrative data repositories in Ontario for research purposes. In Ontario >99% of residents are eligible for universal health care coverage through the Ontario Health Insurance Plan (OHIP). This OHIP number is encrypted and transformed into a unique identification number which can be used to link across multiple health administrative datasets held at ICES. For this validation study, each child in each reference sleep database was confidentially and directly linked to their health administrative data using their OHIP number (CHEO cohort) or probabilistically linked using their hospital medical record number and date of birth (Sickkids cohort). Following linkage, individual participants could not be reidentified. The following administrative databases were used: OHIP (contains all outpatient diagnosis and fee codes), the Canadian Institute of Health Information Discharge Abstract Database (CIHI-DAD) (includes all inpatient diagnostic and procedural codes), the National Ambulatory Care Reporting System (NACRS) (includes all emergency department healthcare encounters), the Same Day Surgery database (SDS) (includes all diagnostic surgical encounters), and the Assistive Devices Program database (ADP) (to ascertain receipt of a CPAP/BiPAP device as the majority of these are fully or partially funded through the Ontario government). These datasets were linked using unique encoded identifiers and analyzed at ICES. This data was accessed between 15 Aug 2020–30 Oct 2020 for performance of statistical analysis. Administrative data codes for PSG, AT, OSA, and CPAP/BiPAP that were used to derive case definitions are described in Tables of S2 Table and S3 Table.

### Participants

Information in each clinical sleep database served as the reference gold standard for the designation of children with or without OSA. For both clinical cohorts, a look back period of 5 years was used to ensure that only incident OSA cases were included. That is, all children who had PSG within the 5-year window preceding the first PSG identified during the study observation period for either CHEO or SickKids were considered to represent ‘prevalent’ OSA cases and were excluded from the validation study. Since this process ensured we only included the first diagnostic sleep study, this approach would additionally exclude children with a prior known diagnosis of or previous treatment for OSA (including previous AT, home supplemental oxygen, non-invasive positive pressure ventilation or invasive home mechanical ventilation). All children were observed for a full 2 years after completion of their diagnostic PSG for ascertainment of outcomes that could be identified in administrative data (i.e., codes to indicate AT, CPAP/BiPAP, or OSA diagnosis).

### Defining OSA

**Fig 1:**
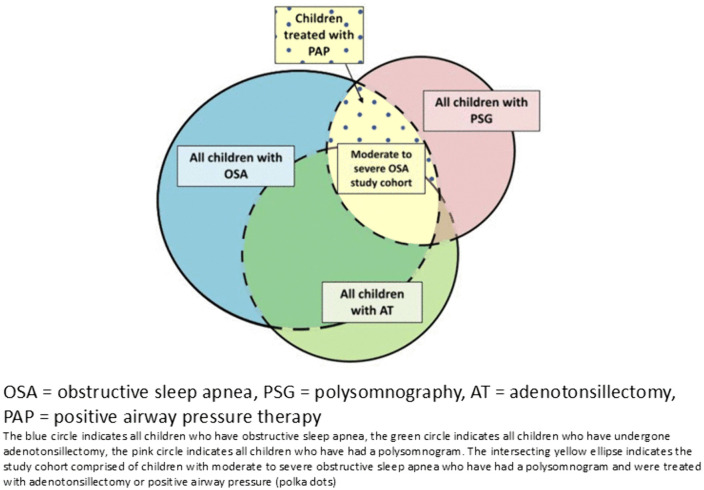
Schematic of children in Ontario with obstructive sleep apnea who would meet a case definition for obstructive sleep apnea using Ontario health administrative data.

For both reference sleep databases, we defined clinically significant OSA in children who underwent a PSG and had a total apnea hypopnea index (AHI) ≥ 5 events/hour, indicating moderate-severe OSA as these are the cases that are the most likely to receive treatment [2]. This limitation to the study cohort was secondary to our assumption that case definitions of OSA that included treatments (i.e., AT, CPAP/BiPAP) would be the most sensitive and specific. A normal PSG was defined as that with an AHI of ≤1. Children with a diagnosis of mixed obstructive/central sleep apnea were included in the OSA cohort, whereas children diagnosed only with central sleep apnea and/or central hypoventilation were not analyzed in this study. Fig 1 depicts a schematic of the subset of Ontario children with OSA that we aimed to capture in this validation study.

### Study procedure and data analysis

We performed descriptive analysis of children included in each database. We then calculated the sensitivity/specificity as well as positive and negative likelihood ratios with 95% confidence limits of Ontario administrative data codes for PSG or AT to assess the accuracy of using these codes for identifying these procedures, using the CHEO sleep database as the gold standard reference. We defined *a* priori that sensitivity/specificity >80% would be considered “high” and >70% would be considered “moderate”; similarly, a positive or negative likelihood ratio >10 or <0.1, respectively would be considered “high”, and near >5 or <0.2, respectively would be “moderate” for the purposes of epidemiologic disease surveillance [23,24]. We then developed various candidate case definitions for OSA using a combination of these verified administrative data codes for varying observation durations of 1–24 months pre- and post- PSG (Table in S4 Table). Case definitions were further informed by discussion with sleep physicians and review of previous studies that have attempted to validate a health administrative diagnosis of OSA in adults [25,26].

Twenty-three different case definitions for OSA (Table in S4 Table) were examined for their sensitivity and specificity as well as positive and negative likelihood ratios against a true diagnosis of OSA among children contained in the ICES-linked reference clinical sleep datasets. Analyses were stratified by age < 10 and ≥10 years old at the time of PSG, to account for differences in pathophysiology and typical treatment approaches between these age groups, which might be captured with varying levels of accuracy in administrative data.

## Results

We achieved 100% linkage of both the Sickkids and CHEO clinical cohorts with the ICES data. There were 760 children included in the SickKids sleep database and 106 were excluded due to duplicates, missing variables, previous PSG during the washout period, missing DOLC or if the child was OHIP ineligible at the time of diagnostic PSG; 654 children remained in the final cohort.

**Fig 2:**
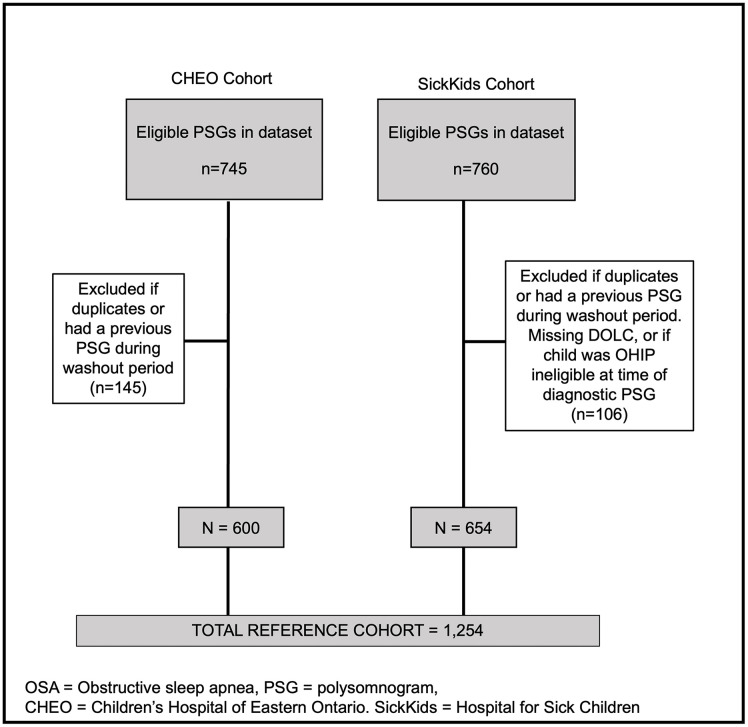
Flow Diagram for CHEO and SickKids clinical cohort creation.

There were 745 PSGs in the CHEO dataset and 145 were excluded due to duplicates, or a previous PSG during the washout period; 600 children remained in the final cohort as shown in Fig 2. Cohort characteristics are described in Table 1.

**Table 1:** Descriptive characteristics of CHEO and SickKids sleep cohorts.

Variable	CHEO (N = 600)		SickKids (N = 654)		Total (N = 1254)	
**Mean Age ± SD** **Median (IQR)**	8.91 ± 4.70 9 (5-13)		6.53 ± 4.65 5 (3-10)		7.67 ± 4.83 7 (3-11)	
**Age category** **n (%)**	<10 years 332 (55.3)	≥ 10 years 268 (44.7)	<10 years 481 (73.5)	≥ 10 years 173 (26.5)	<10 years 813 (64.8)	≥ 10 years 441 (35.2)
**Female** **n (%)**	142 (23.7)	113 (18.8)	216 (33.0)	83 (12.7)	357 (28.5)	196 (15.6)
**Moderate-Severe OSA** **(AHI ≥ 5)** **n (%)**	99 (29.8)	65 (24.3)	122 (25.4)	31 (17.9)	221 (27.2)	96 (21.8)
**Normal** **(AHI ≤ 1)** **n (%)**	140 (42.2)	132 (49.3)	226 (47.0)	97 (56.1)	366 (45.0)	229 (51.9)

CHEO = Children’s Hospital of Eastern Ontario, SD = standard deviation, IQR = interquartile range, AHI = apnea hypopnea index, OSA = obstructive sleep apnea.

A total of 1254 children underwent a first diagnostic PSG during the study period and were included in the study cohort. Of these, 153 children in the SickKids sample and 164 in the CHEO sample had moderate-severe OSA for a total of 317 (25.3%) with moderate-severe OSA in the overall cohort. The proportion of females was 44% with the mean age of the cohort being 7.7 + /- 4.8 years, and 64.8% who were <10 years old at the time of their diagnostic PSG.

Validation of the accuracy of administrative data codes for PSG showed a sensitivity of 0.90 (95%CI 0.87, 0.93) and a specificity of 0.99% (95% CI 0.99, 0.99), (Table in S4 Table) while the sensitivity of administrative data codes for AT was 0.90 (95%CI 0.83, 0.945) and specificity was 0.99 (95%CI 0.98, 0.99) (Table in S5 Table).

Three specific administrative data case definitions for OSA consisting of combinations of PSG plus AT codes, or initiation of positive airway pressure therapy within 0−18 months post PSG, or PSG and a diagnostic code for OSA within 6 months pre, or 0−12, or 0−18, or 0−24 months post PSG performed best with sensitivities, specificities, positive and negative likelihood ratios ranging from 0.62–0.79, 0.80–0.88, 3.82–5.25 and 0.27–0.43, respectively in children <10 years of age. Performance of these same case definitions were less robust in children ≥10 years, with sensitivities, specificities, positive and negative likelihood ratios ranging from 0.28–0.59, 0.85–0.92, 3.48–4.19, 0.48–0.78, respectively (Table 2).

**Table 2:** Validation of best performing health administrative data case definitions of obstructive sleep disordered breathing in Ontario children who underwent polysomnography at a tertiary care pediatric sleep laboratory between 2009 - 2016.

Age	Case Definition	Sensitivity (95% CI)	Specificity (95%CI)	+LR (95%CI)	-LR (95%CI)
**Age < 10 years**	1. PSG + OSA diagnosis 6 months pre or 24 months post PSG	0.72 (0.66, 0.78)	0.85 (0.82, 0.88)	4.75 (3.79, 5.70)	0.33 (0.26, 0.40)
	2. PSG + OSA diagnosis 6 months pre or 12 months post PSG	0.62 (0.55, 0.68)	**0.88** (0.86, 0.91)	**5.25** (4.01, 6.48)	0.43 0.36, 0.51)
	3. PSG + AT/or CPAP/BiPAP within 18 months post PSG/ or OSA diagnosis 6 months pre or 18 months post PSG	**0.79** (0.73, 0.84)	0.80 (0.76, 0.83)	3.82 (3.18, 4.46)	**0.27** (0.20, 0.34)
**Age ≥ 10 years**	1. PSG + OSA diagnosis 6 months pre or 24 months post PSG	0.41 (0.32, 0.50)	0.90 (0.87, 0.93)	**4.19** (2.64, 5.75)	0.66 (0.56, 0.76)
	2. PSG + OSA diagnosis 6 months pre or 12 months post PSG	0.28 (0.20, 0.36)	**0.92** (0.89, 0.95)	3.48 (1.96, 5.00)	0.78 (0.69, 0.87)
	3. PSG + AT/or CPAP/BiPAP within 18 months post PSG/ or OSA diagnosis 6 months pre or 18 months post PSG	**0.59** (0.50, 0.68)	0.85 (0.82, 0.89)	3.99 (2.87, 5.11)	**0.48** (0.37, 0.58)

95% CI = 95% Confidence intervals, LR = likelihood ratio, PSG = polysomnogram, OSA = Obstructive Sleep Apnea, AT = adenotonsillectomy, PAP = positive airway pressure therapy.

Bolded values represent the ‘best’ value for sensitivity, specificity, positive or negative likelihood ratios.

## Discussion

This study describes the first successful attempt to validate case definitions for OSA in children using health administrative data with the development of three case definitions with high specificity and moderate sensitivity, positive and negative likelihood ratios for children with moderate to severe OSA in Canada. Case definitions among children <10 years of age performed particularly well with sensitivity and specificity as high as 79% and 88%, and positive likelihood ratios as high as 5.25, or negative likelihood ratios as low as 0.27, depending on the specific case definition used.

The higher sensitivity of case definitions for OSA in children <10 years of age maybe partially explained by the high frequency of AT used as a treatment in this population, and its accurate capture in health administrative data, as a surgical procedure. On the contrary, OSA case definitions in older children were more reliant on codes indicating use of CPAP/BiPAP. While CPAP/BiPAP is typically the recommended first line therapy in older children with moderate-severe OSA, due to difficulties with adherence and potential out-of-pocket costs for families without private insurance coverage, this treatment is likely used in a smaller proportion of older children, compared to the proportion of younger children who undergo AT. Furthermore, reporting of certain variables related to dispensing of CPAP/BiPAP previously were not mandatory in the Ontario Assistive Devices Program dataset at the time of this study, which may have led to missing data and underreporting of CPAP/BiPAP use in administrative data. These datasets have since been revised with improved accuracy, suggesting algorithms in older children may now perform better.

Previous studies in adults have similarly demonstrated variable success in identifying adult patients with OSA using administrative data. In one Ontario study of 4353 patients included in a hospital surgical database and linked to administrative data, the authors concluded that none of their case definitions provided a positive likelihood ratio that was high enough to adequately identify patients with OSA, given reliance of these case definitions on prescription of CPAP/BiPAP [25]. Kendzerska *et al.* studied two large cohorts of adult patients (N = 18,585) and used a different approach that modelled probabilities of OSA using additional patient characteristics including demographics and comorbidities [26]. This prior study identified case definitions with sensitivities of 59–60%, and specificities of 87–88%, and positive and negative likelihood ratios of 4.5–5.0 and 0.5 respectively, leading them to conclude that identifying patients with moderate to severe OSA could be done with high specificity and good discriminative ability, but at the expense of low sensitivity [26].

The case definitions for identifying children with OSA in the current study are comparable to case definitions routinely used in Ontario health administrative data for identifying children with asthma. The most frequently used case definition for asthma in Ontario and used at ICES has a sensitivity of 89% and specificity of 72% in children. This suggests that the ability to identify OSA from Ontario administrative data using the case definitions presented in this study will enable robust population-based research in children with OSA emulating decades of research in children and adults with asthma [27–30].

The greatest limitation of the current study is that the case definitions derived here represent only a fraction of all children with OSA. This is due to our strict inclusion of only children who had undergone diagnostic PSG. There are a much larger number of children with unconfirmed OSA in Ontario who are on waiting lists for diagnostic PSG. In a previous study performed by our group, we observed that >85% of 27,800 children <10 years of age who underwent treatment with AT for suspected OSA did not have a prior PSG [31]. There is a larger still population of children with both undiagnosed and untreated OSA who may simply improve over time, or may not get diagnosed until adulthood and would not be captured in this study by our case definitions. As availability of PSG increases, the proportion of children with OSA captured by these case definitions may also increase. This study additionally focused on children with clinically significant OSA (i.e., moderate to severe) as this is the population that is more often treated. It should be noted that one of the three most sensitive and specific case definitions for OSA found in this validation study included treatments for OSA (i.e., AT or CPAP/BiPAP), and as such, future epidemiologic studies using this definition would be limited to children with treated OSA.

An additional limitation pertains to generalizability as we restricted our sample to children with PSG performed at 2 pediatric tertiary care centers. Since the completion of data collection for this study, a number of additional, though smaller, pediatric sleep laboratories have opened across the province of Ontario where children with less severe disease and/or fewer comorbidities are studied. Furthermore, since 2009, adult sleep laboratories are increasingly performing PSG in children 10–18 years and a few in children as young as 7 years [31]. As such, it is unclear whether the OSA case definitions tested in the current study would still perform well when applied across the province and further broader validation may need to be considered in future. Such validation would have to take into account that PSG scoring criteria differ between adolescents 13 and older and adults, compared to children <13 years old. While there is ongoing effort to ensure quality control and use of pediatric standards when younger children are studied in adult sleep labs, this is an evolving process [32]. Finally, the standards for scoring and interpreting sleep studies in children as per the AASM changed in 2012, which could have implications on the quality of our reference sleep databases. However, as the SickKids cohort did include children with PSGs in 2016 (and these were scored per updated AASM standards) and comprises more than 25% of our reference cohort sample size, this reassures us as to the validity of this study’s findings.

Despite these limitations, our study establishes validated case definitions for pediatric OSA that accurately identify children with OSA within Ontario’s health administrative data. Applying these case definitions in future research will enable the creation of more true and representative cohorts thereby improving the accuracy and interpretability of population-based studies. These validated definitions can now serve as a foundation for addressing clinical and epidemiological questions, such as identifying predictors of downstream health outcomes (e.g., emergency department visits, development of comorbid conditions) and evaluating responses to different OSA treatments over time or across sub-populations [33]. By improving case ascertainment, this study strengthens the bridge between research and clinical practice, allowing for more meaningful translation of research findings into evidence-informed care for children with OSA.

## Conclusion

We report the first successful development and validation of pediatric case definitions using population-based Ontario health administrative data for identifying children with OSA that are sensitive and specific, particularly among children <10 years of age. These case definitions can be used in future epidemiologic studies to understand the natural history, predictors, and outcomes of children with moderate-severe OSA, with opportunity to confidently translate research findings to clinical care. Refined case definitions may be needed for research and clinical application in older age groups.

## Supporting information

S1 Table The REporting of studies Conducted using Observational Routinely collected health Data (RECORD) Statement. The RECORD statement – checklist of items, extended from the STROBE statement, which should be reported in observational studies using routinely collected health data.
(DOCX)

S2 Table OHIP, CIHI and additional Ontario health administrative codes used in OSA case definitions. (DOCX)

S3 Table List of candidate health administrative data case definitions for obstructive sleep apnea. ^a^PSG and ^b^AT were defined by codes in S2 Table. ^c^ CPAP/bipap were defined as per ADP database codes in S3 Table. ^d^ OSA diagnosis codes included ANY ICD-9 or ICD-10 code for OSA as listed in S2 Table. *These 3 case definitions had the highest sensitivity/specificity and were applied to the full validation cohort (see case definitions 1, 2, 3 in Table 2).
(DOCX)

S4 Table Estimated 95% confidence intervals for validation of polysomnography codes. (DOCX)

S5 Table Estimated 95% confidence intervals for validation of adenotonsillectomy codes. (DOCX)
