# Supplementary material for: Validation of a health administrative definition of obstructive sleep apnea in children in Ontario, Canada
Source: PLoS One. 2026 Apr 27;21(4):e0347148. doi: 10.1371/journal.pone.0347148 (PMC13119826; doi:10.1371/journal.pone.0347148)
Supplement: S2 Table — (DOCX) [file pone.0347148.s002.docx]

**Table S2. OHIP, CIHI and additional Ontario health administrative codes used in OSA case definitions**

| Procedure/  Diagnosis | OHIP Billing Code | Description | CIHI codes (in DAD or NACRS-SDS) |
| --- | --- | --- | --- |
| PSG | J696, J896  J690, J890  J689, J889  J697, J897  J898  J899  J990,  J895, J695 | Diagnostic Polysomnogram – Inital study  Diagnostic Polysomnogram  Therapeutic Polysomnogram (split, CPAP titration)  Diagnostic Polysomnogram (repeat)  Diagnostic Polysomnogram (incomplete) <1 h  Diagnostic Polysomnogram (incomplete) 1-4 h  Diagnostic Polysomnogram (incomplete) >4 h  Therapeutic Polysomnogram | AN.59^^ |
| AT | S063  S065 | Tonsillectomy (includes adenoidectomy), Adenoidectomy only | CIHI codes less reliable (date often missing) so not used |
| OSA |  | Sleep Apnea, obstructed  Other sleep apnea  Unspecified sleep apnea  Sleep Apnea | ICD-10: G4730, G4738  ICD-9: 780.5  327.2 |
| PAP | ADP Codes and descriptions | | |

| Sleep disordered breathing from ADP. Respiratory Database* | - RESP_ELIG_Q1 (Eligibility Q1 - applicant has a diagnosis of OSA and has the presence of symptoms without therapy and the absence of symptoms with therapy) = “Yes” **OR** - DEVICE1_REQD = “Continuous Positive Airway Pressure” **OR** - DEVICE2_REQD = “Bi-level Positive Airway Pressure” **OR** - DEVICE3_REQD = “Auto-titrating Positive Airway Pressure” - KEEP DATE: Date of APAP/CPAP/BPAP prescription: VENDOR_SIGNED_DATE, if missing use PHYSICIAN_SIGNED_DATE (if more than one application per patient, restrict to first application) |
| --- | --- |
| PAP from ADP. Ventilatory | - BPAP_ST_REQD = “Y” OR - VENTILATOR_REQD VENTILATOR="Y" |

PSG: polysomnogram; any combination of these codes was used to identify children who underwent a PSG;

AT: adenotonsillectomy; any combination of these codes was used to identify children who underwent AT;

OSA: Obstructive sleep apnea; any of these ICD-10 or ICD-9 codes was used to identify children with possible OSA; ADP: Assistive devices program dataset; PAP positive airway pressure

* These codes were used to identify children eligible or prescribed positive airway pressure therapy
