# Supplementary material for: Validation of a health administrative definition of obstructive sleep apnea in children in Ontario, Canada
Source: PLoS One. 2026 Apr 27;21(4):e0347148. doi: 10.1371/journal.pone.0347148 (PMC13119826; doi:10.1371/journal.pone.0347148)
Supplement: S3 Table — aPSG and bAT were defined by codes in S2 Table. c CPAP/bipap were defined as per ADP database codes in S3 Table. d OSA diagnosis codes included ANY ICD-9 or ICD-10 code for OSA as listed in S2 Table. *These 3 case definitions had the highest sensitivity/specificity and were applied to the full validation cohort (see case definitions 1, 2, 3 in Table 2). (DOCX) [file pone.0347148.s003.docx]

|  | **Case Definitions** |
| --- | --- |
| 1 | PSG^a^ + subsequent AT^b^ <6 months post PSG |
| 2 | PSG + subsequent AT <12 months post PSG |
| 3 | PSG + subsequent AT <18 months post PSG |
| 4 | PSG + subsequent AT <24 months post PSG |
| 5 | PSG + subsequent prescription for CPAP/BiPAP^c^ <6 months post PSG |
| 6 | PSG + subsequent prescription for CPAP/BiPAP <12 months post PSG |
| 7 | PSG + subsequent prescription for CPAP/BiPAP <18 months post PSG |
| 8 | PSG + subsequent prescription for CPAP/BiPAP <24 months post PSG |
| 9 | PSG + subsequent AT (within 0-24 months) + any OSA^d^ diagnosis code listed in DAD or SDS/NACRS at any time from PSG date up to and including date of AT surgery admission |
| 10 | PSG + any subsequent code within 0-24 months for Sleep apnea, obstructed (i.e. ICD-10 G4730 in DAD/NACRS/SDS) |
| 11 | PSG + any subsequent code within 0-24 months for Other sleep apnea (i.e. ICD-10 G4738 in DAD/NACRS/SDS) |
| 12 | PSG + any subsequent code within 0-24 months for Any ICD-10 code for OSA in DAD/NACRS/SDS (i.e. G4730 or G4738) |
| 13 | PSG + AT within <18 months post OR PSG + CPAP/biPAP <18 months post OR PSG + OSA code in <24 months post (i.e. combination of 3 OR 7 OR 12) |
| 14* | PSG + any subsequent code within 6 months BEFORE or 0-24 months AFTER for any ICD-10 code for OSA in DAD/NACRS/SDS (i.e. G4730 or G4738) |
| 15 | PSG + subsequent second PSG within 12 months afterwards |
| 16 | PSG + subsequent second PSG within 12 months afterwards AND prescription for CPAP/BiPAP <12 months post PSG |
| 17 | PSG + subsequent second PSG within 12 months afterwards OR prescription for CPAP/BiPAP <12 months post PSG |
| 18 | PSG + any subsequent code within 0-12 months for Any ICD-10 code for OSA in DAD/NACRS/SDS (i.e. G4730 or G4738) |
| 19 | PSG + any subsequent code within 0-18 months for Any ICD-10 code for OSA in DAD/NACRS/SDS (i.e. G4730 or G4738) |
| 20* | PSG + any subsequent code within 6 months BEFORE or 0-12 months AFTER for Any ICD-10 code for OSA in DAD/NACRS/SDS (i.e. G4730 or G4738) |
| 21 | PSG + any subsequent code within 6 months BEFORE or 0-18 months AFTER for Any ICD-10 code for OSA in DAD/NACRS/SDS (i.e. G4730 or G4738) |
| 22 | PSG + AT within <18 months post OR PSG + CPAP/biPAP <18 months post OR PSG + any subsequent code within 0-18 months for Any ICD-10 code for OSA in DAD/NACRS/SDS (i.e. G4730 or G4738) |
| 23* | PSG + AT within <18 months post OR PSG + CPAP/biPAP <18 months post OR PSG + any subsequent code within 6 months BEFORE or 0-18 months AFTER for Any ICD-10 code for OSA in DAD/NACRS/SDS (i.e. G4730 or G4738) |

**Table D.**

| **Parameter** | **Estimate** | **95% confidence intervals** |
| --- | --- | --- |
| Sensitivity (%) | 90.13 | 86.70, 92.76 |
| Specificity (%) | 99.90 | 99.88, 99.92 |
| Positive Predictive Value (%) | 77.70 | 73.6, 81.4 |
| Negative Predictive Value (%) | 99.96 | 99.95, 99.97 |

**Table E.**

| **Parameter** | **Estimate** | **95% confidence intervals** |
| --- | --- | --- |
| Sensitivity (%) * | 90.29 | 83.04, 94.64 |
| Specificity (%) * | 99.08 | 97.89, 99.61 |
| Positive Predictive Value (%) | 97.41 | 88.61, 97.80 |
| Negative Predictive Value (%) | 99.96 | 97.71, 99.02 |
