# Supplementary material for: Validation of a health administrative definition of obstructive sleep apnea in children in Ontario, Canada
Source: PLoS One. 2026 Apr 27;21(4):e0347148. doi: 10.1371/journal.pone.0347148 (PMC13119826; doi:10.1371/journal.pone.0347148)
Supplement: S4 Table — (DOCX) [file pone.0347148.s004.docx]

| **Parameter** | **Estimate** | **95% confidence intervals** |
| --- | --- | --- |
| Sensitivity (%) | 90.13 | 86.70, 92.76 |
| Specificity (%) | 99.90 | 99.88, 99.92 |
| Positive Predictive Value (%) | 77.70 | 73.6, 81.4 |
| Negative Predictive Value (%) | 99.96 | 99.95, 99.97 |
