# Supplementary material for: Validation of a health administrative definition of obstructive sleep apnea in children in Ontario, Canada
Source: PLoS One. 2026 Apr 27;21(4):e0347148. doi: 10.1371/journal.pone.0347148 (PMC13119826; doi:10.1371/journal.pone.0347148)
Supplement: S5 Table — (DOCX) [file pone.0347148.s005.docx]

| **Parameter** | **Estimate** | **95% confidence intervals** |
| --- | --- | --- |
| Sensitivity (%) * | 90.29 | 83.04, 94.64 |
| Specificity (%) * | 99.08 | 97.89, 99.61 |
| Positive Predictive Value (%) | 97.41 | 88.61, 97.80 |
| Negative Predictive Value (%) | 99.96 | 97.71, 99.02 |
